# Comparisons of Prediction Models of Quality of Life after Laparoscopic Cholecystectomy: A Longitudinal Prospective Study

**DOI:** 10.1371/journal.pone.0051285

**Published:** 2012-12-28

**Authors:** Hon-Yi Shi, Hao-Hsien Lee, Jinn-Tsong Tsai, Wen-Hsien Ho, Chieh-Fan Chen, King-Teh Lee, Chong-Chi Chiu

**Affiliations:** 1 Department of Healthcare Administration and Medical Informatics, Kaohsiung Medical University, Kaohsiung, Taiwan; 2 Department of Surgery, Chi Mei Medical Center, Liouying, Taiwan; 3 Department of Computer Science, National Pingtung University of Education, Pingtung, Taiwan; 4 Emergency Department, Kaohsiung Municipal United Hospital, Kaohsiung, Taiwan; 5 Department of Health Business Administration, Meiho University, Pigntung, Taiwan; 6 Division of Hepatobiliary Surgery, Department of Surgery, Kaohsiung Medical University Hospital, Kaohsiung, Taiwan; 7 Department of General Surgery, Chi Mei Medical Center, Tainan, Taiwan; 8 Taipei Medical University, Taipei, Taiwan; 9 Chia Nan University of Pharmacy and Science, Tainan, Taiwan; Queen's University Belfast, United Kingdom

## Abstract

**Background:**

Few studies of laparoscopic cholecystectomy (LC) outcome have used longitudinal data for more than two years. Moreover, no studies have considered group differences in factors other than outcome such as age and nonsurgical treatment. Additionally, almost all published articles agree that the essential issue of the internal validity (reproducibility) of the artificial neural network (ANN), support vector machine (SVM), Gaussian process regression (GPR) and multiple linear regression (MLR) models has not been adequately addressed. This study proposed to validate the use of these models for predicting quality of life (QOL) after LC and to compare the predictive capability of ANNs with that of SVM, GPR and MLR.

**Methodology/Principal Findings:**

A total of 400 LC patients completed the SF-36 and the Gastrointestinal Quality of Life Index at baseline and at 2 years postoperatively. The criteria for evaluating the accuracy of the system models were mean square error (MSE) and mean absolute percentage error (MAPE). A global sensitivity analysis was also performed to assess the relative significance of input parameters in the system model and to rank the variables in order of importance. Compared to SVM, GPR and MLR models, the ANN model generally had smaller MSE and MAPE values in the training data set and test data set. Most ANN models had MAPE values ranging from 4.20% to 8.60%, and most had high prediction accuracy. The global sensitivity analysis also showed that preoperative functional status was the best parameter for predicting QOL after LC.

**Conclusions/Significance:**

Compared with SVM, GPR and MLR models, the ANN model in this study was more accurate in predicting patient-reported QOL and had higher overall performance indices. Further studies of this model may consider the effect of a more detailed database that includes complications and clinical examination findings as well as more detailed outcome data.

## Introduction

Laparoscopic cholecystectomy (LC) is among the most common operations performed by general surgeons. Accurately predicting quality of life (QOL), a standard outcome measure after LC, is important when selecting treatment modality and when allocating scarce medical resources [1]–[3].

Regression analysis, one of the most widely used multivariate analysis methods, assumes linear relationships between independent and dependent variables. However, studies show that changes in biomedical variables are often non-linear [4]–[11]. The major classifier methods use support vector machines (SVMs) to solve classification problems by constructing hyperplanes in a multidimensional space that separates cases of different class labels. However, SVMs have also proven effective for solving regression problems because they can handle multiple continuous variables [4]–[6]. Gaussian process regression (GPR) is a kernel-based nonlinear regression technique for using either a kernel or a covariance function for implicitly transforming the data into a high-dimensional reproduction of kernel Hilbert space. The method has proven effective for solving various regression problems [7], [8]. Artificial neural networks (ANNs) are complex and flexible nonlinear systems with properties not found in other modeling systems. These properties include robust performance in dealing with noisy or incomplete input patterns, high fault tolerance, and the capability to generalize from the input data [9]–[11]. The computational power of an ANN is derived from the distributed nature of its connections. The ANN model is a well established data mining algorithm that is widely used in various fields, from engineering to biomedical science [9]–[11].

Although many outcome-predicting models have been developed using conventional statistical procedures, their application at the individual level is hampered by the high interdependence of the clinical variables involved, which potentially may interact with each other and have reciprocal enhancing effects [12], [13]. Three major limitations of this algorithm are (1) the inability to capture interactions of the disease, (2) the inability to capture the process dynamics, and (3) the very large confidence interval in individual risk assessment. Hence, conventional statistical approaches have intrinsic limitations in handling this complex nonlinear information [14]–[16].

Gholipour et al compared ANNs with linear discrimination analysis in terms of their accuracy in predicting conversion of LC to open surgery [14]. They concluded that ANNs that consider the preoperative health characteristics of patients have superior prediction performance compared to discriminant analysis models. Another retrospective analysis of the prevalence of gallbladder disease and its risk factors by Liew et al compared logistic regression and ANN in terms of their accuracy in predicting conversion of LC to open surgery in obese patients [15]. Again, ANN models significantly outperformed the LR models in predicting the risk factors and prevalence of gallbladder disease and gallstone development in obese patients on the basis of multiple variables related to laboratory and pathological features. In Eldar et al, a comparison of logistic regression, linear discriminant analysis and ANN models in predicting conversion of LC to open surgery again showed that ANN-based models are relatively more effective and practical for predicting successful LCs and their conversion [16].



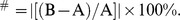



Despite their contribution to the growing understanding of LC surgery outcomes, previous studies of LC outcome have had major shortcomings [12], [17], [18]. Few studies of LC outcome have used longitudinal data for more than two years. Moreover, no studies have considered group differences in factors other than outcome such as age and nonsurgical treatment. Additionally, almost all published articles agree that the essential issue of the internal validity (reproducibility) of ANN, SVM, GPR and multiple linear regression (MLR) models has not been adequately addressed. Therefore, the primary aim of this study was to validate the use of ANN models in predicting patient-reported QOL after LC surgery, and the secondary aim was to compare the predictive capability of ANNs with that of SVM, GPR and MLR models.

## Materials and Methods

### Ethics statement

All patients who had undergone LC performed between May, 2007 and June, 2009 by any surgeons practicing at two tertiary academic hospitals in southern Taiwan were surveyed by the SF-36 and the Gastrointestinal Quality of Life (GIQLI) instruments. Ethical approval was provided by Institutional Review Board of the Kaohsiung Medical University Hospital (KMUH-IRB-960169).

### Study population

Patients provided written informed consent. Patients with cognitive impairment (n = 1), severe organ disease (n = 4) or psychiatric disease (n = 1) were excluded. Of the 518 eligible subjects who gave written consent and were enrolled in the study at baseline, twelve were excluded due to conversion of LC to open cholecystectomy (OC), and 106 were excluded because they did not undergo postoperative assessments. All 400 of the remaining LC subjects completed the preoperative and 2-year postoperative assessments.

### Instruments and measurements

The SF-36 (Chinese version) was administered to measure QOL outcomes, and the score was used as a dependent variable. As described in the literature, the physical component summary scale (PCS) and mental component summary scale (MCS) were calculated using norm-based scoring methods to compare QOL in the study population with that of the general Taiwan population [19]. A PCS or MCS value of 50 was considered average for the general Taiwan population. Both PCS and MCS have been widely adopted and were used in the present study to provide an overall QOL index and for further study of longitudinal changes in generic measures as a whole [20].

The GIQLI is recognized as a valid and reliable instrument for measuring functional status, especially in patients undergoing cholecystectomy [13]. Each of its thirty-six items is scored from 0 to 4 with a higher score indicating better health status, and the total GIQLI score ranges from 0 to 144. A Chinese version of the GIQLI has demonstrated validity [2], [13].

The following patient data obtained by records review and questionnaire interview were tested as independent variables in this study: age, gender, body mass index (BMI), education, Charlson co-morbidity index (CCI) score, marital status, previous abdominal surgery, surgical factors, patient referral source, current alcohol or tobacco use, preoperative functional status, operating time, American Society of Anesthesiologist (ASA) score, current complications, operation time, length of stay (LOS) and re-hospitalization within 30 days.

### System model development

The factors used in the MLR model to predict long-term QOL of LC patients included both demographic and clinical characteristics. The MLR model can be formulated as the following linear equation:
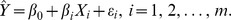
where 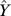 is the actual output value, 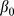 is the intercept, 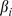 is the model coefficient parameter, 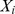 is the independent or input variable, 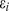 is the random error, and *m* is the number of variables.

The SVM model employs non-linear mapping to transform the original training data into higher-dimensional data and searches for the linear optima that define a hyperplane within the new dimension [4]. With appropriate non-linear mapping to a sufficiently high dimension, a decision boundary can separate data into two classes [4]. In the SVM model, this decision boundary is defined by support vectors and margins.

The GPR applies a Bayesian approach to nonlinear regression. The Bayesian paradigm provides probabilistic modeling of nonlinear regression. The Bayesian approach to regression specifies a priori probabilities of the parameters to be estimated, and it computes the maximum a posteriori probabilities given the observed data samples. Unlike non-Bayesian schemes, which typically choose a single parameter based on a specified criterion, the Bayesian probabilistic model obtains both the optimal estimated function and the covariance associated with the estimation. Therefore, the Bayesian paradigm provides more information about the estimated parameters compared to non-Bayesian methodology. The GPR is a memory-based method of storing some or all of the training data for use in testing. Therefore, GPR can be quickly trained, which improves the efficiency of the massive-training methodology [7].

The ANN model used in this study was a standard feed-forward, back-propagation neural network with three layers: an input layer, a hidden layer and an output layer. The multilayer perceptron (MLP) network is an emerging tool for designing special classes of layered feed-forward networks [21]. Its input layer consists of source nodes, and its output layer consists of neurons; these layers connect the network to the outside world. In addition to these two layers, the MLP usually has one or more layers of neurons referred to as hidden neurons because they are not directly accessible. The hidden neurons extract important features contained in the input data.

### Statistical analysis

The dataset was divided randomly into two sets, one set of 320 cases (80% of the overall dataset) for training the model and another set of eighty cases for testing the model. The model was built using the training set. Demographic and clinical characteristics were the independent variables, and the outcome (QOL) was the dependent variable. The SVM, GPR, MLR and ANN models were then tested using the eighty cases in the testing dataset.

The model fit and prediction accuracy of the system models were measured in terms of mean square error (MSE) and mean absolute percentage error (MAPE), respectively. The MSE, which is computed between the desired and predicted values and then averaged across all data, is used as an indicator of goodness of fit. The MAPE indicates the average deviation from the desired value and is usually expressed as a percentage [22]. The prediction accuracy of a model is considered excellent if its MAPE value is lower than 10%. Values between 10% and 20%, between 20% and 50%, and higher than 50% are considered indicators of high, average, and low prediction accuracy, respectively [22]. The formulas for calculating MSE and MAPE are 
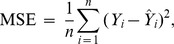



and
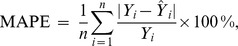
where *n* is the number of observations, 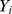 is the desired (target) value of the *i* ^th^ observation, and 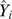 is the actual output value of the *i* ^th^ observation.

The change rates are also given. The optimal number of neurons in the hidden layer and the activation functions are iteratively determined by comparing the MSE index of the output error among several neural networks. The network training process continues as long as training and test errors decrease. That is, training stops when the training error rate and test error rate no longer change or when they begin increasing. The prediction accuracy of the model is then judged by computing the MAPE value. The change rate is also used to compare model performance between the training and test sets. This criterion is used to calculate the difference in MSE index between the test and the training sets so that the better model can be identified. Absolute value was defined as [(the MSE value from test set – the MSE value from training set)/(the MSE value from training set)] ×100%. The lower the change rate and the lower the MSE value are, the better the model performs.

The unit of analysis in this study was the individual LC surgery patient. The data analysis was performed in several stages. Firstly, continuous variables were tested for statistical significance by one-way analysis of variance (ANOVA), and categorical variables were tested by Fisher exact analysis. Univariate analyses were applied to identify significant predictors (P<0.05). Secondly, STATISTICA 10.0 (StatSoft, Tulsa, OK) software was used to construct the MLP network model, the SVM model, the GPR model and the MLR model of the relationship between the identified predictors and QOL. Finally, sensitivity analysis was performed to assess the importance of variables in the fitted models. To simplify the training process, key variables were introduced, and unnecessary variables were excluded. A global sensitivity analysis was also performed to assess the relative significance of input parameters in the system model and to rank the variables in order of importance. The global sensitivity of the input variables against the output variable was expressed as the ratio of the network error (variable sensitivity ratios, VSR) with a given input omitted to the network error with the input included. A ratio of 1 or lower indicates that the variable degrades network performance and should be removed.

## Results

Table 1 shows the patient characteristics in this study. The mean age of the study population was (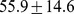) years. The average CCI was 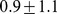, and 57.3% of the patients were female. Furthermore, Table 2 shows the coefficients for total GIQLI score, PCS score, and MCS score obtained by the training set in the MLR model. The selected variables included in the MLR models were age (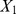), CCI (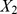), gender (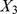), previous abdominal surgery (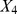), current complications (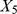), operation time (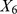), and preoperative functional status (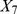). All selected variables were statistically significant (P<0.05) (Table 3). Additionally, in forty runs of the data using an 80%–20% random split, total GIQLI score (Appendix S1), PCS score (Appendix S2) and MCS score (Appendix S3) after surgery did not significantly differ between the training set and testing set.

**Table 1:** Demographic and clinical characteristics of analyzed subjects (N = 400).*

Variables		N (*%*)
Age, years		55.9±14.6
Body mass index, kg/m^2^		24.8±3.8
Duration of symptoms, months		11.8±27.9
Charlson co-morbidity index score		0.9±1.1
Gender	Male	171 (42.7%)
	Female	229 (57.3%)
Education	No formal education/ Primary school	138 (34.5%)
	Junior high school	57 (14.3%)
	Senior high school	121 (30.2%)
	College	84 (21.0%)
Marital status	Single	37 (9.3%)
	Married	363 (90.7%)
Previous abdominal surgery	Yes	129 (32.3%)
	No	271 (67.7%)
Surgical factors	Symptomatic gallstones	252 (63.0%)
	Acute cholecystitis with gallstones	148 (37.0%)
Patient referral source	Outpatient department	303 (75.8%)
	Emergency department	97 (24.2%)
Current drinker	Yes	49 (12.3%)
	No	351 (87.7%)
Current smoker	Yes	68 (17.0%)
	No	332 (83.0%)
Length of stay, days		2.7±2.6
Re-hospitalization within 30 days	Yes	12 (3.0%)
	No	388 (97.0%)
		
Current complications	Yes	27 (6.7%)
	No	373 (93.3%)
Operation time, minutes		85.8±44.1
American Society of Anesthesiologists score		2.1±0.4
Preoperative physical component summary score		48.6±9.3
Preoperative mental component summary score		36.2±20.8
Preoperative total gastrointestinal quality of life index score		102.8±22.5

* Values are means ± standard deviations.

**Table 2:** Coefficients of significant variables for total gastrointestinal quality of life index (GIQLI) score, physical component summary (PCS) score and mental component summary (MCS) score in linear regression model after surgery.

	Total GIQLI score		PCS score		MCS score	
Variables	Coefficients	P value	Coefficients	P value	Coefficients	P value
Age	−0.028	<0.001	−0.018	0.005	−0.027	0.004
Charlson co-morbidity index	−0.714	<0.001	−0.174	<0.001	−0.283	<0.001
Gender (female vs. male)	0.748	0.004	0.694	0.002	0.249	<0.001
Previous abdominal surgery (no vs. yes)	3.766	0.008	1.764	<0.001	0.188	0.002
Current complications (yes vs. no)	5.559	<0.001	1.601	<0.001	−2.020	<0.001
Operation time	−0.031	0.007	−0.014	0.008	−0.004	0.018
Pre-operative functional status	0.084	<0.001	0.034	<0.001	0.069	<0.001

**Table 3:** The mean and standard deviation (SD) of total gastrointestinal quality of life index (GIQLI) score, physical component summary (PCS) score and mental component summary (MCS) score after surgery in forty runs of the data with an 80%–20% random split.

	Training set (320 cases)	Testing set (80 cases)	
Subscales	Mean ± SD	Mean ± SD	P value
Total GIQLI score	122.7±4.9	119.9±4.7	0.171
PCS score	50.5±4.8	50.3±4.9	0.660
MCS score	53.2±6.0	55.0±6.6	0.069

Table 4 shows the three-layer networks and number of support vectors of total GIQLI score, PCS score and MCS score in ANN and SVM models. The ANN-based approaches provided the 3-layer networks and the relative weights of neurons used for predicting QOL. The activation functions of logistic sigmoid and hyperbolic tangent were used in each neuron of the hidden layer and output layer, respectively.

**Table 4:** Three-layer networks and number of support vectors for total gastrointestinal quality of life index (GIQLI) score, physical component summary (PCS) score and mental component summary (MCS) score in artificial neural network (ANN) and support vector machine (SVM) models.

Subscales	ANN-based model*	SVM-based model#
Total GIQLI score	7-4-1	134
PCS score	7-5-1	88
MCS score	7-4-1	140

* Values are for input layer-hidden layer-output layer.

# Values are numbers of support vectors.

Table 5 compares the QOL predictions obtained by the ANN, the SVM, the GPR and the MLR models for the training set and the test set. For predicting QOL, the ANN model had relatively larger change rates and MSE values in the test set with the exception of MSE for the testing set at year 2. That is, the ANN model had better QOL prediction capability. Compared to the other three models, the GPR model had smaller MSE and MAPE values in the training set. In the testing set, however, the GPR model had relatively larger MSE and MAPE values and relatively larger change rates for predicting QOL. The poor predictive performance of the GPR in the testing set may have resulted from overfitting to the training data. Apparently, the ANN model also outperformed the SVM model, the GPR model and the MLR model in terms of predictive accuracy. Most MAPE values obtained by the ANN model were lower than 10%, which indicated the excellent accuracy of the ANN in predicting QOL.

**Table 5:** Comparison of multiple linear regression (MLR), support vector machine (SVM), Gaussian process regression (GPR) and artificial neural network (ANN) models in predicting total gastrointestinal quality of life index (GIQLI) score, physical component summary (PCS) score and mental component summary (MCS) score.

Indices	Models	Training set (A)	Testing set (B)	Change rate^#^
Total GIQLI score				
MSE	MLR	82.14	74.73	9.0%
	SVM	76.50	74.52	2.6%
	GPR	1.3×10^−23^	1038	7.9×10^27^%
	ANN	65.55	54.42	16.9%
MAPE	MLR	4.7%	6.1%	-
	SVM	5.3%	5.3%	-
	GPR	1.5×10^−12^%	20.2%	
	ANN	4.2%	4.7%	-
PCS score				
MSE	MLR	19.91	17.48	12.2%
	SVM	23.39	22.68	3.1%
	GPR	14.94	46.66	212.3%
	ANN	18.62	15.65	15.9%
MAPE	MLR	6.3%	6.0%	-
	SVM	6.8%	6.2%	-
	GPR	4.4%	10.7%	
	ANN	5.8%	5.1%	-
MCS score				
MSE	LR	86.68	80.82	6.7%
	SVM	62.60	60.01	4.1%
	GPR	30.42	87.82	188.7%
	ANN	66.31	58.61	11.6%
MAPE	LR	13.9%	10.9%	-
	SVM	9.7%	10.0%	-
	GPR	8.3%	15.8%	
	ANN	8.6%	6.4%	-

MSE = mean square error, MAPE = mean absolute percentage error.

The training set was also used to calculate the variable sensitivity ratios (VSR) for the ANN model. Table 6 presents the VSR values for the outcome variable (QOL) in relation to the three most influential variables. In the ANN model, preoperative functional status (VSR 1.38) was the most influential (sensitive) parameter in terms of its effects on total GIQLI score, PCS score, and MCS score (VSR 1.38, 1.15, and 1.07, respectively). All VSR values exceeded one, indicating that the network performs better when all variables are considered.

**Table 6:** Global sensitivity analysis of artificial neural network (ANN) model in predicting total gastrointestinal quality of life index (GIQLI) score, physical component summary (PCS) score and mental component summary (MCS) score.*

	Rank 1^st^	Rank 2^nd^	Rank 3^rd^
ANN model	Variable sensitivity ratios	Variable sensitivity ratios	Variable sensitivity ratios
Total GIQLI score	*X*_7_	*X*_2_	*X*_4_
	1.38	1.24	1.14
PCS score	*X*_7_	*X*_4_	*X*_3_
	1.15	1.06	1.02
MCS score	*X*_7_	*X*_5_	*X*_4_
	1.07	1.02	1.02

* Age (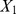), Charlson co-morbidity index score (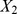), gender (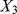), previous abdominal surgery (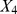), current complications (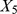), operation time (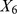), preoperative functional status (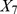).

Table 7 compares the MAPE values obtained by ANN, SVM, GPR and MLR models. Compared to the SVM model, GPR model and the MLR model, the ANN model consistently obtained lower MAPE values for total GIQLI score (4.7% versus 20.8%), PCS score (6.4% versus 12.3%), and MCS score (10.6% versus 20.6%).

**Table 7:** Comparison of mean absolute percentage error (MAPE) in total gastrointestinal quality of life index (GIQLI) score, physical component summary (PCS) score and mental component summary (MCS) score predicted by multiple linear regression (MLR), support vector machine (SVM), Gaussian process regression (GPR), and artificial neural network (ANN) models in forty new data sets.

Models	MAPE
Total GIQLI score	
MLR model	6.2%
SVM model	6.0%
GPR model	20.8%
ANN model	4.7%
PCS score	
MLR model	7.7%
SVM model	7.8%
GPR model	12.3%
ANN model	6.4%
MCS score	
MLR model	14.7%
SVM model	11.8%
GPR model	20.6%
ANN model	10.6%

## Discussion

This study confirmed that, compared to the SVM model, the GPR model and the MLR model, the ANN model is significantly more accurate in predicting QOL (P<0.001). To the best of our knowledge, this study is the first to use ANNs for analyzing predictors of QOL after LC surgery. This model was tested against actual outcomes obtained by a neural network model, a support vector machine model and a linear regression model constructed using identical inputs. We also showed that, given the same number of demographic and clinical inputs and the same two outcome measures, the predictive accuracy of ANN is superior to that of SVM, GPR and MLR.

Recently, SVM, GPR and ANN models have been used for non-linear modeling in many fields, particularly bioinformatics [4]–[11]. Although the efficacy of SVM and GPR models is well established in the field of machine learning, its performance in surgical outcome prediction and prognosis has not been measured. The ANNs are adaptive models that use a dynamic approach to analyzing the risk of outcomes. That is, they perform bottom-up computation by modifying their internal structures in relation to a functional objective (i.e., the model is generated by the data it analyzes). Despite their incapability to deal with missing data, ANNs can simultaneously process numerous variables and can consider outliers and nonlinear interactions among variables. Unlike standard statistical tests, ANNs effectively manage complexity even when samples sizes are small and when ratios between variables and records are unbalanced. In this respect, ANNs avoid the dimensionality problem and can achieve a predictive accuracy superior to those of SVM, GPR and MLR. To ensure a sufficiently robust basis for network training, the present study used a large and homogeneous dataset comprising all demographic and clinical variables shown to affect patient-reported QOL in previous linear regression models [10], [11].

Piaggi and colleagues demonstrated that ANN models can accurately predict weight loss in obese women treated by laparoscopic adjustable gastric banding. Their integrated multidisciplinary approach showed that ANN may be a valuable tool for selecting the best candidates for surgery [23]. Segal and colleagues compared ANN with a multiple linear regression model in terms of accuracy in predicting several different functional outcome scores at 1 year after traumatic brain injury [24]. The predictive accuracy of their sophisticated linear models was comparable to that of ANNs. Recently, Salvatore and colleagues combined multiple linear regressions with artificial neural networks to predict how relationships among lower urinary tract symptoms, anatomical findings, and baseline characteristics affect outcome in women with pelvic organ prolapse. They also found that ANNs are valuable instruments for improving understanding of complex biological models [25].

The ANN approach developed in this study extends the predictive range of the linear regression model by replacing identity functions with nonlinear activation functions. The approach is apparently superior to linear regression for describing systems. The ANNs may be trained with data acquired in various clinical contexts and can consider local expertise, racial differences, and other variables with uncertain effects on clinical outcome. The analysis need not be limited to clinical parameters. Other potentially useful variables could be tested to improve the predictive value of the model. The proposed ANN architecture with MLP can also include more than one dependent variable and can perform a non-linear transformation between dependent variables. Future studies may evaluate how other demographic or clinical characteristics affect the proposed architecture.

In ANNs, overfitting occurs when a model describes random error instead of the underlying relationship. Data overfitting is indicated by an increasing testing error concurrent with a steadily decreasing training error. The model with the best predictive performance and the best data fit is that in which testing error is at the global minimum [26]. Based on the above rule for obtaining a fitted model while avoiding overfitting, this study used the testing set as the controlling criterion for determining when to stop training. Additionally, the testing data were not included in the training data.

Throughout this two-year follow-up study, the best single predictor of QOL subscale scores was preoperative functional status, which is consistent with reports that preoperative functional scores are the best predictors of postoperative QOL [2], [13]. Therefore, effective counseling is essential for apprising patients of expected post-surgery impairments. If QOL outcomes are considered benchmarks, then preoperative functional status, which is a major predictor of postoperative outcome, is crucial. Patients should also be advised that their postoperative QOL might depend not only on the success of their operations, but also on their preoperative functional status.

Post-surgery QOL may be related to surgical risk. For example, Quintana et al. [27] suggested that men with low surgical risks are more likely to be diagnosed with complicated cholelithiasis compared to men with high surgical risks. Since men with low surgical risks are more likely to experience complicated cholelithiasis, they have greater potential for improvement in QOL. This suggests that gender differences in QOL outcomes may result from gender differences in the treatment of complicated presentations, i.e., in terms of QOL, men may derive a greater benefit compared to women because they have a greater potential for complications and thus a greater potential for improvement. Another possible but untested explanation is gender differences in health care received. Some authors also suggest that patient values and the reporting of health status also differ by gender [18], [27]. Another factor is age. This study confirmed previous findings that QOL improvement after LC surgery is inversely related to age [18], [27].

Additionally, a recent study indicates that, compared to patients in early stages of a disease, patients in advanced stages not only tend to have more co-morbidities, they also tend to have less social support [2]. Notably, the CCI score in the current study was inversely related to QOL, which is consistent with the reported association between increased comorbidity and poor postcholecystectomy QOL [28]–[30].

Although all research questions were satisfactorily addressed, several limitations are noted. First, this study collected data for LC surgery patients who had been under the supervision of four surgeons in four different medical centers, each of whom had performed the highest volume of LC surgery procedures in his respective hospital during the previous years. This sample selection procedure ensured that patient outcome data would not be affected by surgeons with limited experience. By focusing the analysis on procedures performed by these four surgeons, the results of this study are more representative of all LC patients compared to one analyzing those performed by a single surgeon. However, a notable limitation is that the first patient in the prospective patient cohort was enrolled in 2007. Therefore, depending on their inclusion date, some surveyed patients had a longer follow-up than others did, which may have caused selection bias. Nonetheless, in most QOL subscales, the characteristics of subjects who continuously participated throughout this 2-year study did not significantly differ from those of subjects who died or dropped out during the study (data not shown).

## Conclusions

Compared with the SVM model, the GPR model and the MLR model, the ANN model in the study was more accurate in predicting patient-reported QOL and had higher overall performance indices. The global sensitivity analysis also showed that preoperative functional status is the most important predictor of total GIQLI score, PCS score and MCS score after LC surgery. The predictors analyzed in this study could be addressed in preoperative and postoperative health care consultations to educate candidates for LC surgery in the expected course of recovery and expected functional outcomes. Further studies of this model with differential evolution [31] may consider the effect of a more detailed database that includes complications and clinical examination findings as well as more detailed outcome data. Hopefully, the model will evolve into an effective adjunctive clinical decision making tool.

## Supporting Information

Appendix S1 **Forty data sets used for comparing predictions of total gastrointestinal quality of life index (GIQLI) score.**
(DOC)

Appendix S2 **Forty new data sets used for comparing predictions of physical component summary (PCS) score.**
(DOC)

Appendix S3 **Forty new data sets used for comparing predictions of mental component summary (MCS) score.**
(DOC)
