# Supplementary material for: Comparisons of Prediction Models of Quality of Life after Laparoscopic Cholecystectomy: A Longitudinal Prospective Study
Source: PLoS One. 2012 Dec 28;7(12):e51285. doi: 10.1371/journal.pone.0051285 (PMC3532431; doi:10.1371/journal.pone.0051285)
Supplement: Appendix S1 — Forty data sets used for comparing predictions of total gastrointestinal quality of life index (GIQLI) score. (DOC) [file pone.0051285.s001.doc]

**Appendix 1. Forty data sets used for comparing predictions of total gastrointestinal quality of life index (GIQLI) score.**

| $X_{1}$ | $X_{2}$ | $X_{3}$ | $X_{4}$ | $X_{5}$ | $X_{6}$ | $X_{7}$ | GIQLI score |
| --- | --- | --- | --- | --- | --- | --- | --- |
| 80 | 0 | 0 | 0 | 0 | 65 | 81 | 120.69 |
| 68 | 1 | 0 | 0 | 0 | 105 | 117 | 116.00 |
| 86 | 1 | 1 | 1 | 0 | 135 | 95 | 127.00 |
| 37 | 2 | 0 | 0 | 0 | 90 | 112 | 136.00 |
| 79 | 1 | 0 | 1 | 0 | 85 | 89 | 120.69 |
| 58 | 3 | 0 | 1 | 0 | 75 | 117 | 120.69 |
| 81 | 2 | 0 | 1 | 0 | 120 | 66 | 120.69 |
| 36 | 0 | 0 | 0 | 0 | 115 | 82 | 120.69 |
| 55 | 1 | 0 | 0 | 0 | 55 | 129 | 128.00 |
| 55 | 0 | 1 | 1 | 0 | 50 | 108 | 120.69 |
| 47 | 2 | 0 | 1 | 0 | 90 | 110 | 120.69 |
| 61 | 2 | 1 | 0 | 0 | 65 | 105 | 120.69 |
| 60 | 0 | 1 | 0 | 0 | 85 | 121 | 134.00 |
| 71 | 1 | 0 | 1 | 0 | 40 | 133 | 114.00 |
| 68 | 1 | 1 | 0 | 0 | 45 | 119 | 124.00 |
| 27 | 1 | 1 | 1 | 0 | 110 | 107 | 120.69 |
| 51 | 1 | 1 | 1 | 1 | 65 | 103 | 120.69 |
| 44 | 0 | 1 | 1 | 0 | 105 | 85 | 132.00 |
| 41 | 2 | 0 | 0 | 0 | 80 | 80 | 90.00 |
| 83 | 1 | 1 | 1 | 1 | 205 | 98 | 120.69 |
| 29 | 0 | 1 | 1 | 0 | 45 | 111 | 114.00 |
| 56 | 0 | 0 | 1 | 0 | 50 | 91 | 126.00 |
| 47 | 0 | 1 | 1 | 0 | 75 | 112 | 130.00 |
| 57 | 3 | 1 | 0 | 0 | 45 | 117 | 132.00 |
| 74 | 0 | 0 | 0 | 0 | 60 | 119 | 74.00 |
| 55 | 0 | 1 | 0 | 0 | 45 | 104 | 130.00 |
| 48 | 0 | 1 | 1 | 0 | 50 | 113 | 116.00 |
| 75 | 1 | 0 | 1 | 0 | 70 | 132 | 120.69 |
| 60 | 3 | 1 | 1 | 0 | 35 | 130 | 120.69 |
| 56 | 0 | 1 | 1 | 0 | 105 | 84 | 120.69 |
| 60 | 0 | 1 | 0 | 0 | 75 | 93 | 123.00 |
| 60 | 0 | 1 | 1 | 0 | 45 | 111 | 119.00 |
| 88 | 0 | 1 | 1 | 0 | 50 | 95 | 120.69 |
| 59 | 0 | 1 | 1 | 0 | 75 | 102 | 121.00 |
| 38 | 1 | 1 | 0 | 0 | 85 | 112 | 125.00 |
| 44 | 0 | 1 | 1 | 0 | 40 | 123 | 126.00 |
| 46 | 1 | 0 | 0 | 0 | 40 | 115 | 120.69 |
| 49 | 0 | 1 | 1 | 0 | 30 | 125 | 120.69 |
| 53 | 0 | 0 | 0 | 0 | 80 | 106 | 120.69 |
| 46 | 0 | 1 | 1 | 0 | 45 | 75 | 128.00 |

^*^Age ($X_{1}$), Charlson co-morbidity index score ($X_{2}$), Gender ($X_{3}$), Previous abdominal surgery ($X_{4}$), Current complications ($X_{5}$), Operation time ($X_{6}$), Preoperative functional status ($X_{7}$)
