# Supplementary material for: Comparisons of Prediction Models of Quality of Life after Laparoscopic Cholecystectomy: A Longitudinal Prospective Study
Source: PLoS One. 2012 Dec 28;7(12):e51285. doi: 10.1371/journal.pone.0051285 (PMC3532431; doi:10.1371/journal.pone.0051285)
Supplement: Appendix S2 — Forty new data sets used for comparing predictions of physical component summary (PCS) score. (DOC) [file pone.0051285.s002.doc]

**Appendix 2. Forty new data sets used for comparing predictions of physical component summary (PCS) score.**

| $X_{1}$ | $X_{2}$ | $X_{3}$ | $X_{4}$ | $X_{5}$ | $X_{6}$ | $X_{7}$ | PCS score |
| --- | --- | --- | --- | --- | --- | --- | --- |
| 80 | 0 | 0 | 0 | 0 | 65 | 29.44 | 51.03 |
| 68 | 1 | 0 | 0 | 0 | 105 | 44.60 | 54.50 |
| 86 | 1 | 1 | 1 | 0 | 135 | 20.14 | 54.02 |
| 37 | 2 | 0 | 0 | 0 | 90 | 48.04 | 55.77 |
| 79 | 1 | 0 | 1 | 0 | 85 | 60.30 | 51.03 |
| 58 | 3 | 0 | 1 | 0 | 75 | 39.32 | 51.03 |
| 81 | 2 | 0 | 1 | 0 | 120 | 39.80 | 51.03 |
| 36 | 0 | 0 | 0 | 0 | 115 | 58.84 | 51.03 |
| 55 | 1 | 0 | 0 | 0 | 55 | 59.37 | 54.15 |
| 55 | 0 | 1 | 1 | 0 | 50 | 51.91 | 51.03 |
| 47 | 2 | 0 | 1 | 0 | 90 | 45.11 | 51.03 |
| 61 | 2 | 1 | 0 | 0 | 65 | 36.76 | 51.03 |
| 60 | 0 | 1 | 0 | 0 | 85 | 48.31 | 53.14 |
| 71 | 1 | 0 | 1 | 0 | 40 | 47.87 | 51.49 |
| 68 | 1 | 1 | 0 | 0 | 45 | 49.57 | 55.92 |
| 27 | 1 | 1 | 1 | 0 | 110 | 41.08 | 51.03 |
| 51 | 1 | 1 | 1 | 1 | 65 | 40.66 | 51.03 |
| 44 | 0 | 1 | 1 | 0 | 105 | 44.36 | 53.86 |
| 41 | 2 | 0 | 0 | 0 | 80 | 51.62 | 23.82 |
| 83 | 1 | 1 | 1 | 1 | 205 | 54.13 | 51.03 |
| 29 | 0 | 1 | 1 | 0 | 45 | 52.74 | 53.45 |
| 56 | 0 | 0 | 1 | 0 | 50 | 50.81 | 55.41 |
| 47 | 0 | 1 | 1 | 0 | 75 | 49.95 | 55.37 |
| 57 | 3 | 1 | 0 | 0 | 45 | 41.53 | 51.57 |
| 74 | 0 | 0 | 0 | 0 | 60 | 38.60 | 62.06 |
| 55 | 0 | 1 | 0 | 0 | 45 | 41.85 | 53.19 |
| 48 | 0 | 1 | 1 | 0 | 50 | 55.00 | 48.89 |
| 75 | 1 | 0 | 1 | 0 | 70 | 57.13 | 51.03 |
| 60 | 3 | 1 | 1 | 0 | 35 | 49.42 | 51.03 |
| 56 | 0 | 1 | 1 | 0 | 105 | 60.21 | 51.03 |
| 60 | 0 | 1 | 0 | 0 | 75 | 51.54 | 55.64 |
| 60 | 0 | 1 | 1 | 0 | 45 | 44.98 | 54.13 |
| 88 | 0 | 1 | 1 | 0 | 50 | 19.49 | 51.03 |
| 59 | 0 | 1 | 1 | 0 | 75 | 42.09 | 53.55 |
| 38 | 1 | 1 | 0 | 0 | 85 | 42.10 | 54.74 |
| 44 | 0 | 1 | 1 | 0 | 40 | 44.46 | 52.91 |
| 46 | 1 | 0 | 0 | 0 | 40 | 53.06 | 51.03 |
| 49 | 0 | 1 | 1 | 0 | 30 | 47.67 | 51.03 |
| 53 | 0 | 0 | 0 | 0 | 80 | 42.03 | 51.03 |
| 46 | 0 | 1 | 1 | 0 | 45 | 50.97 | 53.46 |

^*^Age ($X_{1}$), Charlson co-morbidity index score ($X_{2}$), Gender ($X_{3}$), Previous abdominal surgery ($X_{4}$), Current complications ($X_{5}$), Operation time ($X_{6}$), Preoperative functional status ($X_{7}$)
