# Supplementary material for: Comparisons of Prediction Models of Quality of Life after Laparoscopic Cholecystectomy: A Longitudinal Prospective Study
Source: PLoS One. 2012 Dec 28;7(12):e51285. doi: 10.1371/journal.pone.0051285 (PMC3532431; doi:10.1371/journal.pone.0051285)
Supplement: Appendix S3 — Forty new data sets used for comparing predictions of mental component summary (MCS) score. (DOC) [file pone.0051285.s003.doc]

**Appendix 3. Forty new data sets used for comparing predictions of mental component summary (MCS) score.**

| $X_{1}$ | $X_{2}$ | $X_{3}$ | $X_{4}$ | $X_{5}$ | $X_{6}$ | $X_{7}$ | MCS score |
| --- | --- | --- | --- | --- | --- | --- | --- |
| 80 | 0 | 0 | 0 | 0 | 65 | 19.30 | 53.81 |
| 68 | 1 | 0 | 0 | 0 | 105 | 57.38 | 57.66 |
| 86 | 1 | 1 | 1 | 0 | 135 | 54.48 | 63.79 |
| 37 | 2 | 0 | 0 | 0 | 90 | 56.43 | 63.08 |
| 79 | 1 | 0 | 1 | 0 | 85 | 19.37 | 53.81 |
| 58 | 3 | 0 | 1 | 0 | 75 | 68.63 | 53.81 |
| 81 | 2 | 0 | 1 | 0 | 120 | 2.16 | 53.81 |
| 36 | 0 | 0 | 0 | 0 | 115 | 10.25 | 53.81 |
| 55 | 1 | 0 | 0 | 0 | 55 | 24.94 | 54.07 |
| 55 | 0 | 1 | 1 | 0 | 50 | 60.75 | 53.81 |
| 47 | 2 | 0 | 1 | 0 | 90 | 62.31 | 53.81 |
| 61 | 2 | 1 | 0 | 0 | 65 | 59.16 | 53.81 |
| 60 | 0 | 1 | 0 | 0 | 85 | 53.97 | 59.29 |
| 71 | 1 | 0 | 1 | 0 | 40 | 61.78 | 53.18 |
| 68 | 1 | 1 | 0 | 0 | 45 | 47.71 | 60.03 |
| 27 | 1 | 1 | 1 | 0 | 110 | 67.92 | 53.81 |
| 51 | 1 | 1 | 1 | 1 | 65 | 32.60 | 53.81 |
| 44 | 0 | 1 | 1 | 0 | 105 | 4.95 | 51.06 |
| 41 | 2 | 0 | 0 | 0 | 80 | 5.32 | 37.72 |
| 83 | 1 | 1 | 1 | 1 | 205 | 42.22 | 53.81 |
| 29 | 0 | 1 | 1 | 0 | 45 | 54.47 | 52.60 |
| 56 | 0 | 0 | 1 | 0 | 50 | 55.59 | 60.07 |
| 47 | 0 | 1 | 1 | 0 | 75 | 28.73 | 55.50 |
| 57 | 3 | 1 | 0 | 0 | 45 | 48.25 | 61.51 |
| 74 | 0 | 0 | 0 | 0 | 60 | 43.07 | 2.55 |
| 55 | 0 | 1 | 0 | 0 | 45 | 0.14 | 63.28 |
| 48 | 0 | 1 | 1 | 0 | 50 | 20.3 | 53.33 |
| 75 | 1 | 0 | 1 | 0 | 70 | 62.83 | 53.81 |
| 60 | 3 | 1 | 1 | 0 | 35 | 57.27 | 53.81 |
| 56 | 0 | 1 | 1 | 0 | 105 | 12.13 | 53.81 |
| 60 | 0 | 1 | 0 | 0 | 75 | 48.81 | 48.23 |
| 60 | 0 | 1 | 1 | 0 | 45 | 58.66 | 56.17 |
| 88 | 0 | 1 | 1 | 0 | 50 | 29.46 | 53.81 |
| 59 | 0 | 1 | 1 | 0 | 75 | 39.39 | 55.92 |
| 38 | 1 | 1 | 0 | 0 | 85 | 50.34 | 63.16 |
| 44 | 0 | 1 | 1 | 0 | 40 | 53.19 | 64.34 |
| 46 | 1 | 0 | 0 | 0 | 40 | 56.36 | 53.81 |
| 49 | 0 | 1 | 1 | 0 | 30 | 65.95 | 53.81 |
| 53 | 0 | 0 | 0 | 0 | 80 | 29.24 | 53.81 |
| 46 | 0 | 1 | 1 | 0 | 45 | 29.78 | 51.88 |

^*^Age ($X_{1}$), Charlson co-morbidity index score ($X_{2}$), Gender ($X_{3}$), Previous abdominal surgery ($X_{4}$), Current complications ($X_{5}$), Operation time ($X_{6}$), Preoperative functional status ($X_{7}$)
